# Spatial patterns and drivers of temperature sensitivity of winter evergreen vegetation on the Tibetan Plateau

**DOI:** 10.3389/fpls.2026.1891108

**Published:** 2026-08-20

**Authors:** Jinxia Lv, Chun Dong, Guangyong Li, Xiaohuang Liu, Rui Zhang, Rong Zhao

**Affiliations:** 1 Chinese Academy of Surveying and Mapping, Beijing, China; 2 Beijing Forestry University, Beijing, China; 3 Laboratory of Coupling Process and Effect of Natural Resources Elements, Ministry of Natural Resources, Beijing, China; 4 State Key Laboratory of Cryospheric Science and Frozen Soil Engineering, Key Laboratory of Remote Sensing of Gansu Province, Heihe Remote Sensing Experimental Research Station, Northwest Institute of Eco-Environment and Resources, Chinese Academy of Sciences, Lanzhou, China

**Keywords:** climate warming, driving factors, evergreen vegetation, temperature sensitivity, Tibetan plateau

## Abstract

Climate warming has enhanced the winter greenness of evergreen vegetation on the Tibetan Plateau over the past 20 years. This response is primarily determined by the temperature sensitivity of vegetation, rather than the absolute extent of warming. However, how these factors shape the spatial patterns of sensitivity of vegetation to temperature (abbreviated as vegetation sensitivity) remains poorly understood. In this study, we analyzed the spatial pattern of vegetation sensitivity and its driving factors by using partial least squares regression, pixel-scale multiple linear regression, zone-level linear mixed models, and random forest model based on the winter normalized vegetation index (NDVI_winter_) and multiple driving factors datasets. We found that the dominant factors influencing winter vegetation greenness are growing-season temperature and precipitation. The 72.5% of pixels exhibited positive vegetation sensitivity, with higher values in the south of the Himalayas and southeast edge. Vegetation sensitivity varied significantly across aridity zones, with the highest and lowest values in humid and arid zones, respectively. Furthermore, mixed forests showed the highest sensitivity. Random Forest analysis revealed that downward shortwave radiation (Ssrd) and the aridity index (AI) may contribute to spatial variability in NDVI_winter_ sensitivity to temperature, while the Ssrd and elevation were the most important environmental factors in humid and arid zones, respectively. Partial dependence plots indicated nonlinear relationships between key driving factors and NDVI_winter_ sensitivity, with precipitation and AI showing negative associations, while mean temperature exhibited a positive trend. The elevation and Ssrd displayed threshold-like responses, with sharp transitions in their influence on vegetation sensitivity. These findings clarify the complex regulatory roles of radiation, water availability, temperature, and topography in shaping the spatial heterogeneity of winter vegetation temperature sensitivity, and improve our understanding of alpine ecosystem responses to climate change.

## 1 Introduction

Vegetation is crucial for climate regulation, by influencing the exchange of energy, water and carbon to control land surface temperature (Bonan, 2008; Piao et al., 2019). The IPCC AR6 report, global mean annual temperature is projected to rise by 2 °C or more by the end of the century, which would increase the vulnerability of ecosystems, especially in climate change sensitive regions (Lee et al., 2023; Wu et al., 2024). Climate warming has altered vegetation growth and distribution patterns (Kelly and Goulden, 2008; Løkken et al., 2020; Wang et al., 2022). The sensitivity of vegetation to temperature, the critical indicators of climate change, determines the pattern of the ecosystem to climate shifts and influences vegetation growth dynamic (Li et al., 2019; Seddon et al., 2016; Wu et al., 2024). Especially, the sensitivity of vegetation to temperature exhibits pronounced spatial heterogeneity, contributed by differences in climate and environmental conditions (Li et al., 2019; Wu et al., 2024, 2017). Therefore, considering climate change intensifies, revealing the spatial distribution of the sensitivity of vegetation to temperature and understanding its driving factors could provide support for future predicting of vegetation dynamics and ecological stability.

The Tibetan Plateau, often referred to as the “Third pole”, exhibits a high sensitivity to global climate change (Kuang and Jiao, 2016; Zhang et al., 2023). In recent decades, the plateau has experienced rapid climate change, with a rate of warming that is twice the global average rate (You et al., 2021). Contributed to increased temperature and precipitation, there has been widespread greening of vegetation on the Tibetan Plateau, characterized by a notable rise in vegetation cover (including evergreen vegetation greening) (Huang et al., 2022; Lv et al., 2024; Zhong et al., 2019), an upward shift in the treelines (Wang et al., 2022), and the expansion of shrublands (Liu et al., 2025a). On the previous study, we found that the greening of winter evergreen vegetation was more dependent on the sensitivity of the vegetation to temperature rather than temperature magnitude (Lv et al., 2024). While there is evidence indicating temporal changes in vegetation sensitivity to temperature on the global scale (Wang et al., 2024a), less attention in the spatial pattern of the sensitivity of evergreen vegetation to temperature on the Tibetan Plateau.

Current approaches to measuring vegetation sensitivity to temperature primarily include linear regression, exponential quantile regression, and composite index methods (Wang et al., 2024a; Wu et al., 2024, 2017). Linear regression methods were widely used for obtaining vegetation sensitivity at the pixel scale (Shen et al., 2022; Wang et al., 2024a), ignoring the time-dependent characteristics of vegetation to climate change. The exponential quantile regression method is designed to characterize extreme responses at different quantiles (Wu et al., 2017). However, the selection of quantiles lacks objective criteria, and sparsity of data in the distribution tails leads to highly unstable estimates. The composite index method bundles multiple processes into a single composite sensitivity index; while this facilitates comparison, it obscures the distinct response mechanisms associated with different climate changes (Chen et al., 2024; Li et al., 2019; Wu et al., 2024). Previous studies have focused on the trends of vegetation sensitivity, as well as the responses of vegetation growth to temperature and precipitation (Chen et al., 2024; Ma et al., 2019; Quetin and Swann, 2017; Wang et al., 2024a; Wu et al., 2024). For instance, the alpine ecosystems of the Qinghai-Tibet Plateau exhibit a strong response to climate fluctuations, with vegetation growth sensitivity generally increasing during 1982-2015 (Wu et al., 2024). Furthermore, the researches on the temperature sensitivity of vegetation phenology has confirmed that regional dry-wet differentiation is the key factor in shaping the spatial pattern of vegetation sensitivity (Du et al., 2019). Although existing studies have revealed changes in vegetation sensitivity and their driving factors, they have primarily focused on vegetation phenology and have typically relied on a single methodological framework. Moreover, differences in the sensitivity of evergreen vegetation across multiple aridity gradients and vegetation types, as well as the coupled driving mechanisms of multiple factors, remain insufficiently investigated.

Therefore, the object of study is to (1) quantify the spatial pattern of sensitivity of winter vegetation greenness to temperature on the Tibetan Plateau during 2000–2022 for pixel scale and across different vegetation and aridity zones; (2) to estimate the main driving factors of spatial differentiation in temperature sensitivity.

## 2 Materials and methods

### 2.1 Study area

The Tibetan Plateau is the most unique geologic-geographic-ecological unit, spanning 26°00′12″ N to 39°46′50″ N and 73°18′52″ E to 104°46′59″ E, with an average elevation of more than 4,000 m. The region of winter evergreen vegetation was determined by multiyear mean of winter NDVI during 2000-2022, with deciduous vegetation and mutation by mining activities carefully excluded from the analysis (Lv et al., 2024). The region is mainly located in the southeast of the plateau, land cover types vary from grassland, shrubs to evergreen forests from eastward (Lv and Zhao, 2025). The elevations are ranged from 84–5811 m, generally lower along the eastern and southeastern margins and increasing along the northwestern direction. The multiyear mean of temperature shows a similar spatial pattern, decreasing from 23 °C to -9 °C with increasing altitude. Multiyear mean of precipitation is highest in the southern part of the Himalayas exceeds 10000 mm and relatively low in the north-east with 79 mm, with a decreasing trend from south-east to north-west.

### 2.2 Data collection and preprocessing

#### 2.2.1 Vegetation greenness

The vegetation greenness was measured by the normalized difference vegetation index (NDVI) data. Winter NDVI has been shown to be useful for monitoring changes in evergreen vegetation greenness (Lv et al., 2024).The NDVI data were obtained from MOD13A1 version 6.1, which has a spatial resolution of 500 m, a temporal resolution of 16 days (Didan, 2021), and a time range from 2000 to 2022. Furthermore, to eliminate the effects of clouds, shadows, and other interfering factors, the monthly maximum values were synthesized, and then the arithmetic mean of the NDVI values from December to February of the next year was calculated as the annual mean of winter NDVI (NDVI_winter_). For additional details on NDVI data preprocessing methodologies, please refer to Lv et al., 2024 (Lv et al., 2024).

#### 2.2.2 Climate data

The climate data including temperature, precipitation and downward shortwave radiation, were obtained from ERA5-Land dataset, with a temporal resolution of 1 hour and a spatial resolution of 0.1° (Muñoz Sabater, 2019). Among available climate products, ERA5 performs well in reproducing the temporal variations in temperature and precipitation on the Tibetan Plateau (Lv et al., 2024; Peng et al., 2023; Sun et al., 2021). The data were resampled to 500 m based on the matching of geographical coordinates. We firstly calculated the annual growing season (May-September) mean temperature (T_GS_) and cumulative precipitation and downward shortwave radiation from 2000 to 2022 and then we obtained the multiyear mean of temperature, precipitation and downward shortwave radiation by the arithmetic mean ways.

#### 2.2.3 Landcover data

The land cover data of 2010 year was obtained from MCD12Q1 version 6.1, with a spatial resolution of 500 m and the classification system is the International Geosphere Biosphere Programme (IGBP), in which land cover types are carefully categorized into 17 different classes (Friedl and Sulla-Menashe, 2022).

#### 2.2.4 Elevation data

To further evaluate the driving factors of sensitivity of vegetation greenness to temperature, the Digital Elevation Model (DEM), soil moisture, and aridity data were also used in the analysis. The DEM data was obtained from Shuttle Radar Topography Mission version v003, and the data have a spatial resolution of 1 arc second, which is approximately 30 m (NASA-JPL, 2013) and then resampled to 500 m by bilinear interpolation method.

#### 2.2.5 Soil moisture data

Soil moisture data were generated through machine learning techniques, leveraging observations from 1,648 stations that monitor soil moisture across 10 distinct layers, as provided by the China Meteorological Administration (CMA) (Shangguan et al., 2022). This analysis incorporated ERA5_Land meteorological forcing data, Leaf Area Index (LAI), land cover classifications, topographical features, and soil properties as covariates. The dataset offers daily-scale soil moisture measurements with a spatial resolution of 1 km, which represents an optimal choice for capturing the spatial heterogeneity of alpine ecosystems across the Tibetan Plateau, covering soil depths from 0 to 100 cm for the period spanning 2000 to 2021. This high-resolution dataset provides unprecedented insights into soil moisture dynamics with remarkable spatial and temporal precision. Then the soil moisture data is resampled to 500 m by nearest neighbor interpolation method. Additionally, we used 0–100 cm soil moisture based on ecological and literature evidence. Globally, 95% of terrestrial plant roots occur within 2 m depth, with a median rooting depth of 0.88 m (Schenk and Jackson, 2002). Qilian juniper, a dominant evergreen species in our region, concentrates most fine roots in 40–100 cm (Wang et al., 2019). Alpine shrubs and grasslands on the Tibetan Plateau also develop shallow root systems largely confined to the upper 1 m (Aroca et al., 2016; Liu et al., 2025b).

#### 2.2.6 Aridity index data

Additionally, the aridity index was derived by calculating the ratio of potential evapotranspiration to precipitation (Equation 1), boasting a spatial resolution of 1 km and an annual temporal resolution (Peng, 2023), and then resampled to 500 m by nearest neighbor interpolation method. The formula is as follows:

(1) AI=pet/pre

where *AI* is annual aridity index, *pet* and *pre* are annual potential evapotranspiration (mm) and annual precipitation (mm). Aridity serves as a quantifiable metric for assessing the dryness level of a region, with higher aridity values indicating reduced atmospheric water vapor content and, consequently, greater arid condition. For the purposes of this study, we utilized the multi-year average aridity index data spanning from 2000 to 2021. According to the AI classification (Peng, 2023), the study region can be broadly categorized into humid (AI< 1), semi-humid (1 ≤ AI< 1.5), semi-arid (1.5 ≤ AI< 4), and arid zones (AI ≥ 4).

### 2.3 Methods

**Figure 1:**
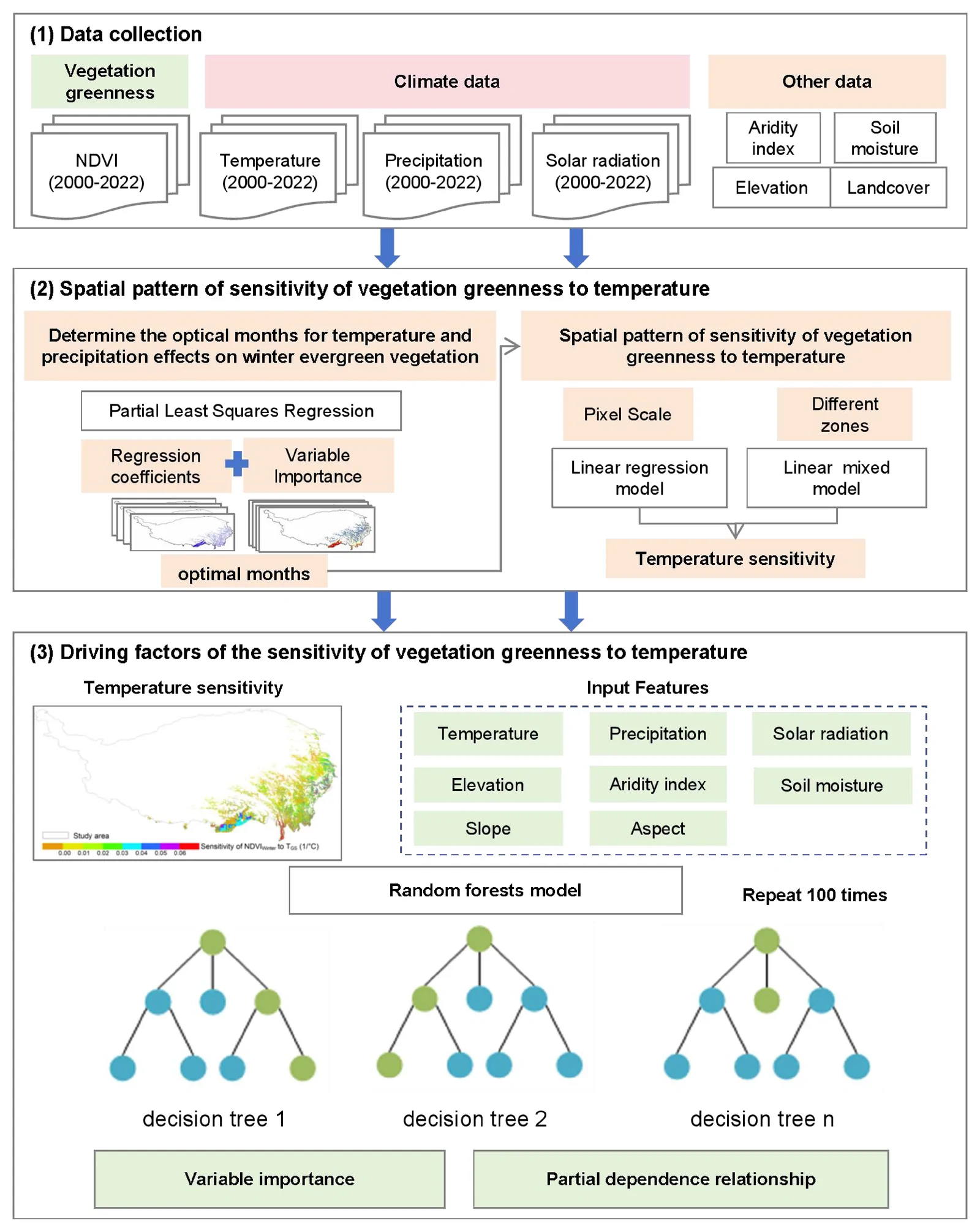
Study flowchart.

Using dataset with consistent spatial resolution, this study implemented a three-stage analytical framework to investigate the spatial pattern for sensitivityof vegetation to temperature and its driving factors. First, the partial least squares regression (PLSR) method was applied to identify the optimal months for climate factors effects on winter NDVI, accounting for multicollinearity among temperature and precipitation variables. Second, pixel-scale multiple linear regression and zone-level linear mixed models (LMM) with pixel ID as random effects were conducted to quantify the sensitivity of winter vegetation to temperature. Third, the random forest model was used to evaluate the relative importance of topographic, climatic, and soil variables in shaping spatial patterns of temperature sensitivity (**Figure 1**).

#### 2.3.1 Determining the optimal months for temperature and precipitation effects on vegetation growth

The Partial Least Squares Regression (PLSR) is a distinctive statistical method that integrates multiple linear regression, correlation analysis, and principal component analysis, and is specifically designed for modeling relationships between multiple dependent variables and multiple independent variables (Geladi and Kowalski, 1986). This approach effectively addresses common problems in ordinary multiple regression, such as limited sample size and multicollinearity among predictor variables. The temperature and precipitation are the most significant factors influencing the growth of vegetation (Huang et al., 2022; Lv et al., 2024) and there is a significant multicollinearity among the climate factors. Therefore, the PLSR method was used to assess the dominant driving factors in vegetation greenness dynamics, which could determine the optimal months for temperature and precipitation effects on winter vegetation greenness.Prior to analysis, all independent and dependent variables were standardized. The optimal number of components was determined using leave-one-out cross-validation by minimizing the predicted residual error sum of squares (PRESS) and evaluating cross-validated. Components were retained if their cross-validated performance exceeded a threshold of 0.0975. Variable importance in the projection (VIP) was calculated to evaluate the relative contribution of each predictor, with VIP > 1 indicating influential variables. PLSR was implemented pixel-scale across the study region to preserve spatial heterogeneity.

#### 2.3.2 Sensitivity of vegetation greenness to temperature calculation

On the basis of the optimal months for temperature and precipitation effects on winter vegetation greenness, we further analysis of the sensitivity of winter vegetation to temperature. We used the linear regression method and linear mixed model (LMM) to calculate the sensitivity of vegetation to temperature at the pixel scale and different zones, respectively (Shen et al., 2022). At the pixel scale, the sensitivity of vegetation can be estimated individually for each pixel by applying a multiple linear regression model between winter NDVI_winter_, mean temperature, and cumulative precipitation over the relevant optimal months (Equation 2). The formula is as follows:

(2) $$\text{y}=\beta_{0}+\beta_{1}\times \mathrm{temp}+\beta_{2}\times \mathrm{pre}+\epsilon$$

where *y* is NDVI_winter_ during 2000-2022, *temp* and *pre* are the mean temperature (°C) and cumulative precipitation (mm) of relevant optimal months, β_0_ is intercept, β_1_ and β_2_ are regression coefficients, ϵ is error term.

The LMM integrates random effects into the framework of linear regression, thereby enabling the differentiation of variations within groups and those between groups (Gumedze and Dunne, 2011; Wu et al., 2026). Therefore, the LMM was used to estimate the vegetation sensitivity to temperature for different vegetation types and aridity zones with pixel ID as random effects.

#### 2.3.3 Quantifying the driving factors of vegetation sensitivity

Random forests model is a nonparametric, ensemble-based machine learning methodology that constructs multiple decision trees from bagging of training samples, to enhance generalization performance through collective inference (Breiman, 2001). The random forest model was robust to multicollinearity and maintains stable predictive performance (Breiman, 2001), which was used to assess the importance of variables for sensitivity of vegetation greenness to temperature. We selected seven driving factors to analyze the variable importance of vegetation sensitivity, including elevation, slope, aspect, multiyear mean of growing season temperature, precipitation and solar radiation, soil moisture, and aridity index. Furthermore, we first separated the data, with 75% for training, and the remaining 25% for validation with 100 decision trees. Then, to ensure the robustness of the results, the random forest model was run 100 times repeatedly, and the average values of the variable importance and partial dependence relationship of vegetation sensitivity were analyzed. The root mean squared error (RMSE) and coefficient of determination (R^2^) were as model performance metrics.

## 3 Results

### 3.1 The optimal months for temperature and precipitation effects on winter evergreen vegetation

**Figure 2:**
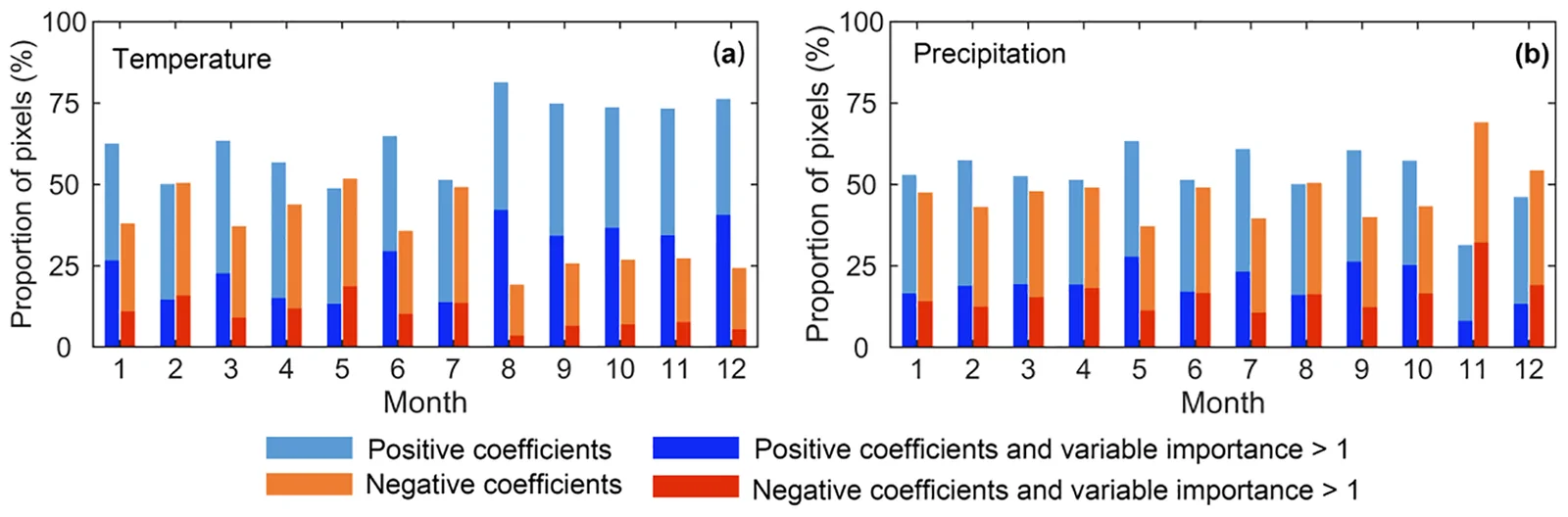
Proportion of pixels for partial least squares regression coefficients of climate factors on winter NDVI during 2000–2021 for monthly mean **(a)** temperature and **(b)** precipitation.

In this study, the PLSR model was employed to analyze the impacts of monthly mean temperature and monthly cumulative precipitation from January to December on NDVI_winter_, and to identify the dominant climatic factors driving the vegetation variation. The August, September and October exhibit the largest proportions of pixels with positive partial least squares regression coefficients between monthly mean temperature and NDVI_winter_; among these months and August has the highest proportion of pixels with variable importance greater than 1 (**Figure 2a**). In contrast, the overall proportion of pixels with positive regression coefficients between monthly cumulative precipitation and NDVI_winter_ is lower than that of temperature (**Figure 2b**). The impacts of precipitation in May, July and September on NDVI_winter_ are stronger than those in other months. Therefore, the climatic factors dominating the variation in NDVI_winter_ in this region are mainly growing-season (from May to September) temperature and precipitation.

Spatially, the influences of August, September and October temperatures on NDVI_winter_ are mainly concentrated in the southeastern and southern parts of the study area (**Supplementary Figures 1**, **2**). The May, September and October precipitation exerts positive effects on NDVI_winter_ are primarily distributed in the northeastern part and eastern edge of the area (**Supplementary Figures 3**, **4**). Temperature and precipitation in other months also affect vegetation growth, but with relatively low contributions. Accordingly, consistent with the period of active vegetation growth, growing-season temperature and precipitation were used for further analysis of the sensitivity of winter vegetation NDVI to temperature.

### 3.2 Spatial pattern of sensitivity of vegetation greenness to temperature

**Figure 3:**
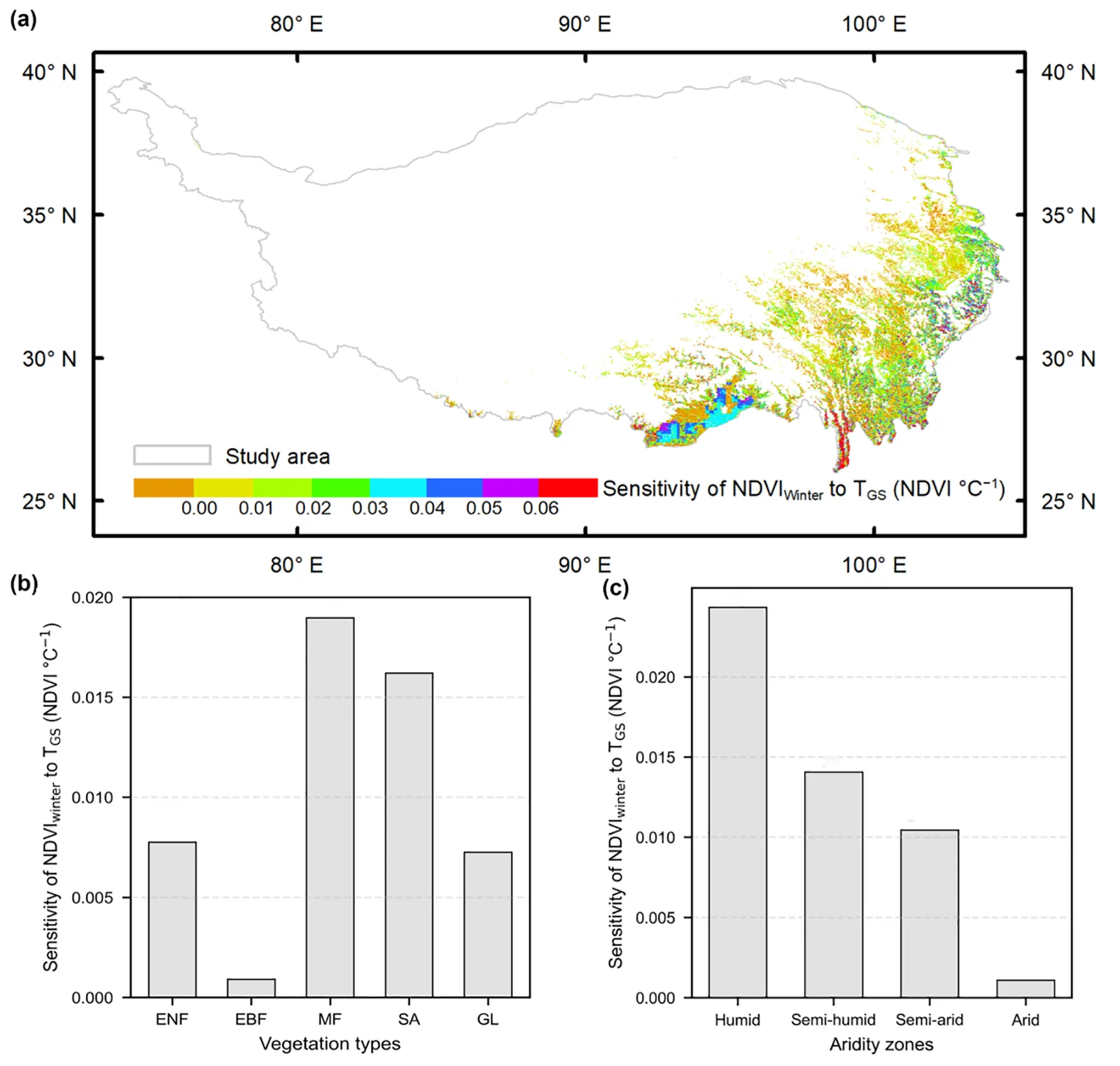
Distribution of the sensitivity of winter NDVI (NDVI_winter_) to growing season temperature (T_GS_). **(a)** Spatial pattern of the sensitivity of NDVI_winter_ to T_GS_, and the variation among different **(b)** vegetation types and **(c)** aridity zones. ENF, Evergreen Needleleaf Forests; EBF, Evergreen Broadleaf Forests; MF, Mixed Forests; SA, Savannas; GL, Grasslands.

The sensitivity of NDVI_winter_ to T_GS_ was mostly positive, accounting for 72.5% of all pixels (19.78% significant, **Supplementary Figure 5**), with high values (> 0.060 NDVI °C^-1^) in south of the Himalayas and southeast edge, suggesting these regions showed higher vegetation sensitivity to temperature (**Figure 3a**). Conversely, the negative value of sensitivity of NDVI_winter_ to T_GS_ was scattered in the southern and eastern parts of the plateau. Furthermore, the vegetation sensitivity to temperature was positive for all vegetation types, indicating that NDVI_winter_ was generally positively regulated by growing-season temperature on the Tibetan Plateau. Notable differences in sensitivity were observed among vegetation types. Specifically, the mixed forests exhibited highest value (0.019 NDVI °C^-1^) of sensitivity of NDVI_winter_ to T_GS_, followed by savannas, while evergreen broadleaf forests were observed with the lowest sensitivity of 0.00091 NDVI °C^-1^ (**Figure 3b**). Similarly, there is significant spatial variation of sensitivity of NDVI_winter_ to T_GS_ during different aridity zones, with highest and lowest sensitivity in humid and arid zones, respectively (0.024 and 0.0011 NDVI °C^-1^), suggesting that vegetation sensitivity to temperature varies significantly across different aridity zones (**Figure 3c**).

### 3.3 Driving factors of the sensitivity of vegetation greenness to temperature

**Figure 4:**
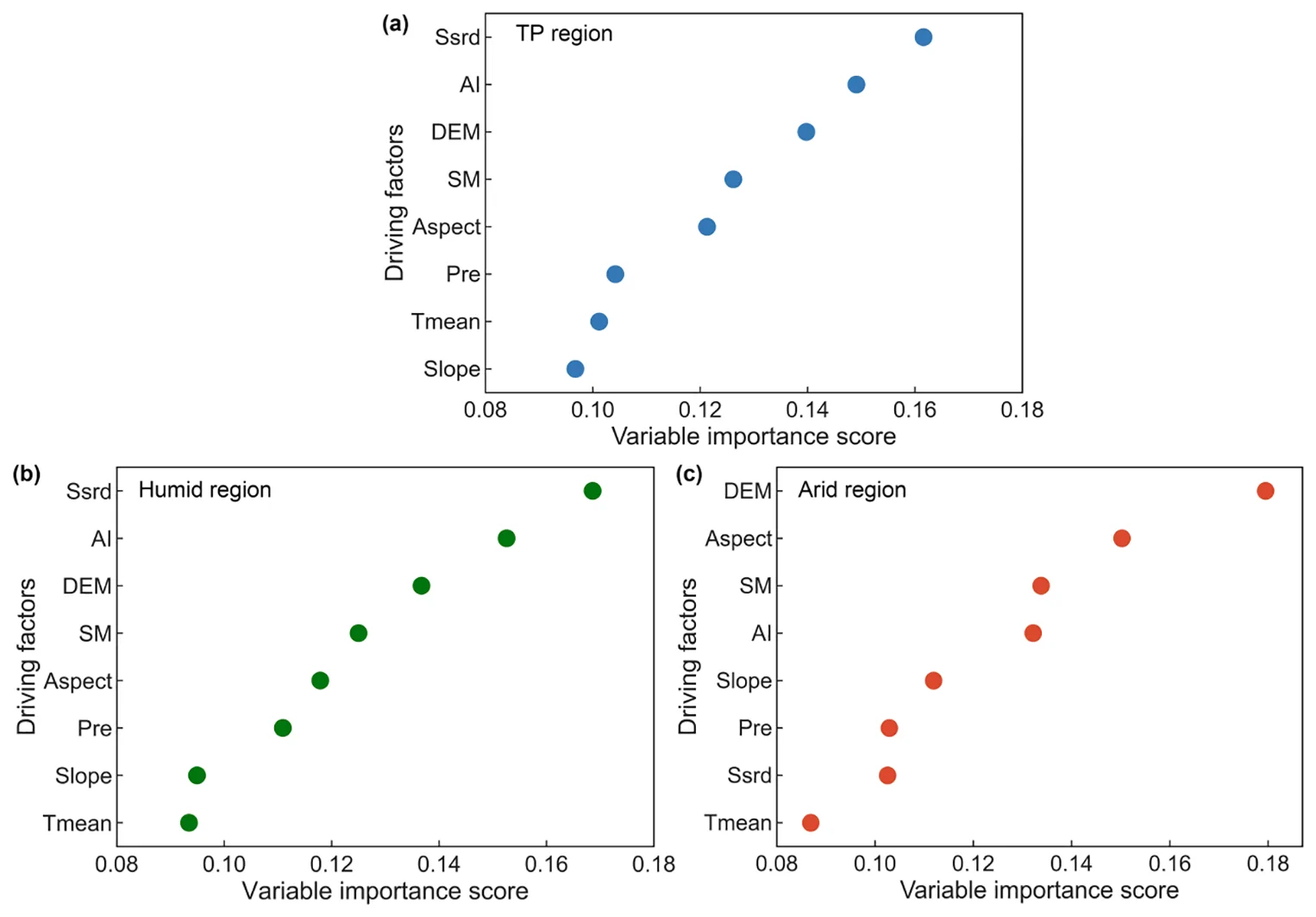
Importance of variable for the sensitivity of winter NDVI to growing season temperature **(a)** for the Tibetan Plateau (TP) region, **(b)** humid region and **(c)** arid region. Ssrd, multiyear mean of cumulative downward shortwave radiation in growing season during 2000–2021; Pre, multiyear mean of cumulative precipitation in growing season during 2000–2021; Tmean, multiyear mean of average temperature in growing season during 2000–2021; AI, multiyear mean of annual aridity index during 2000–2021; SM, multiyear mean of soil moisture of 0–100 cm during 2000–2021.

The Random Forest revealed that the spatial variability in sensitivity of NDVI_winter_ to T_GS_ was predominantly influenced by downward shortwave radiation (Ssrd) and the aridity index (AI) (**Figure 4a**) and the mean RMSE and R^2^ of the model were 0.020 and 0.52 (**Supplementary Figure 6**). Meanwhile, the importance of variable showed low variability of all driving factors, with all coefficients of variation below 0.02 (**Supplementary Table 1**). We also implemented spatial 10-fold cross-validation for further robustness verification. The cross-validated mean R² values was 0.53. The distinct differences were observed in the key driving factors influencing these spatial patterns between humid and arid regions, suggesting region-specific environmental controls on vegetation response to temperature. In humid regions, the spatial variability of NDVI_winter_ sensitivity to T_GS_ was predominantly influenced by climatic factors, with Ssrd and AI exerting the strongest influences (**Figure 4b**) and the mean RMSE and R^2^ of the model were 0.020 and 0.52 (**Supplementary Figure 6**). In contrast, arid regions exhibited a distinct pattern, where topographic factors, particularly DEM and aspect, were the primary determinants of this sensitivity (**Figure 4c**) and the mean RMSE and R^2^ of the model were 0.019 and 0.41 (**Supplementary Figure 6**).

**Figure 5:**
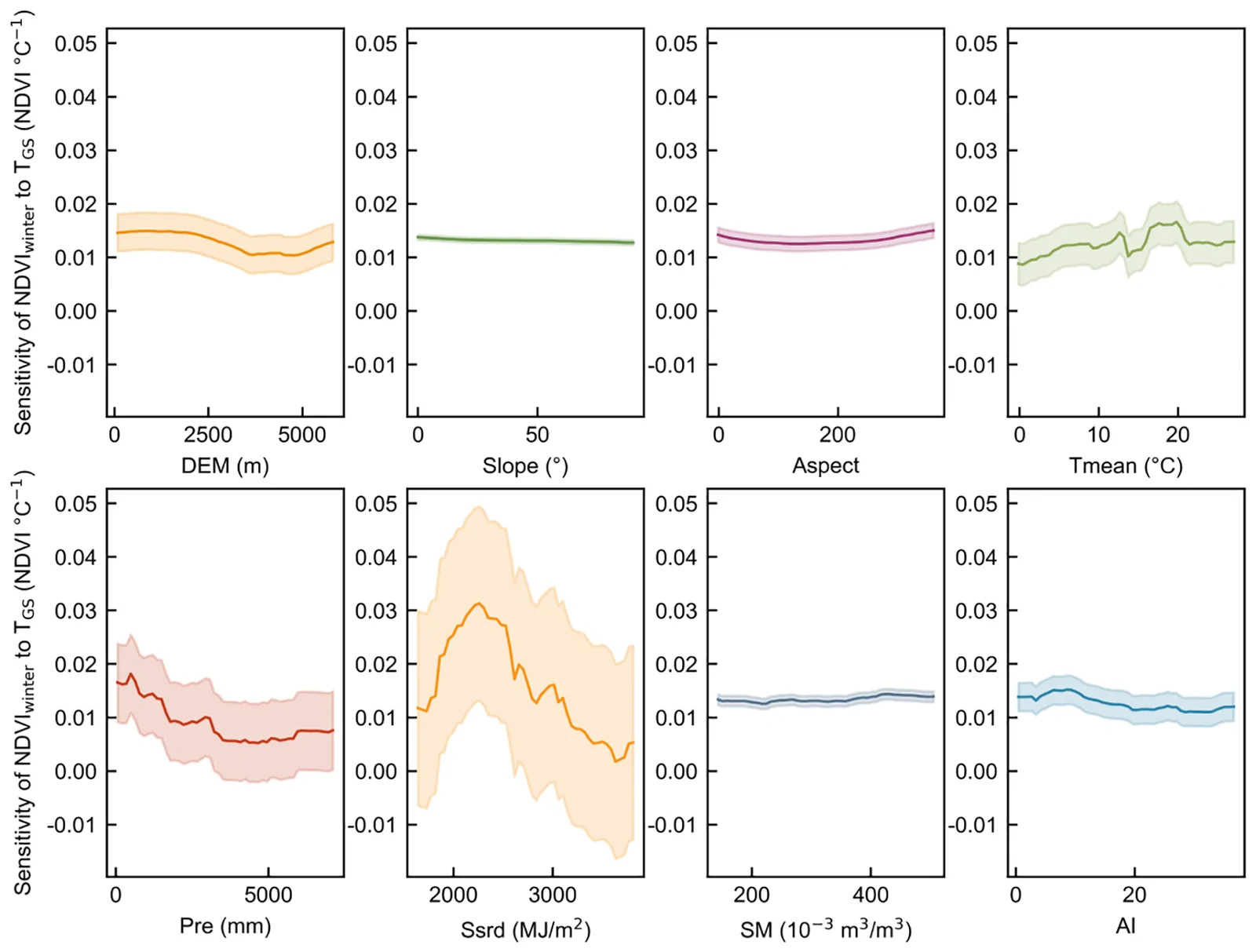
Partial dependency relationship between driving factors and the sensitivity of winter NDVI (NDVI_winter_) to growing season temperature (T_GS_). The shaded area refers to the 95% confidence interval. Ssrd, multiyear mean of cumulative downward shortwave radiation in growing season during 2000–2021; Pre, multiyear mean of cumulative precipitation in growing season during 2000–2021; Tmean, multiyear mean of average temperature in growing season during 2000-2021; AI, multiyear mean of annual aridity index during 2000–2021; SM, multiyear mean of soil moisture of 0–100 cm during 2000–2021.

Partial dependence plots (**Figure 5**) revealed distinct nonlinear relationships between key driving factors and NDVI_winter_ sensitivity to T_GS_. The precipitation (Pre), and AI exhibited negative nonlinear associations with vegetation sensitivity, whereas mean temperature (Tmean) demonstrated a positive nonlinear trend (**Figure 5**). Notably, DEM and Ssrd displayed a threshold-like response, with a sharp transition in its influence on vegetation sensitivity. In 3000 m< DEM< 4000 m, DEM showed a threshold where NDVI_winter_ sensitivity to T_GS_ was lowest; between 2000 MJ/m^2^< Ssrd< 2500 MJ/m^2^, NDVI_winter_ sensitivity to T_GS_ was highest. Additionally, slope exhibited a comparatively weak influence on NDVI_winter_ sensitivity to T_GS_, with vegetation on south-facing slopes showing notably reduced temperature sensitivity compared to other aspects. However, the slope and soil moisture (SM) showed less relation with NDVI_winter_ sensitivity to T_GS_.

### 3.4 The differences in the effects of major environmental factors on temperature sensitivity

**Figure 6:**
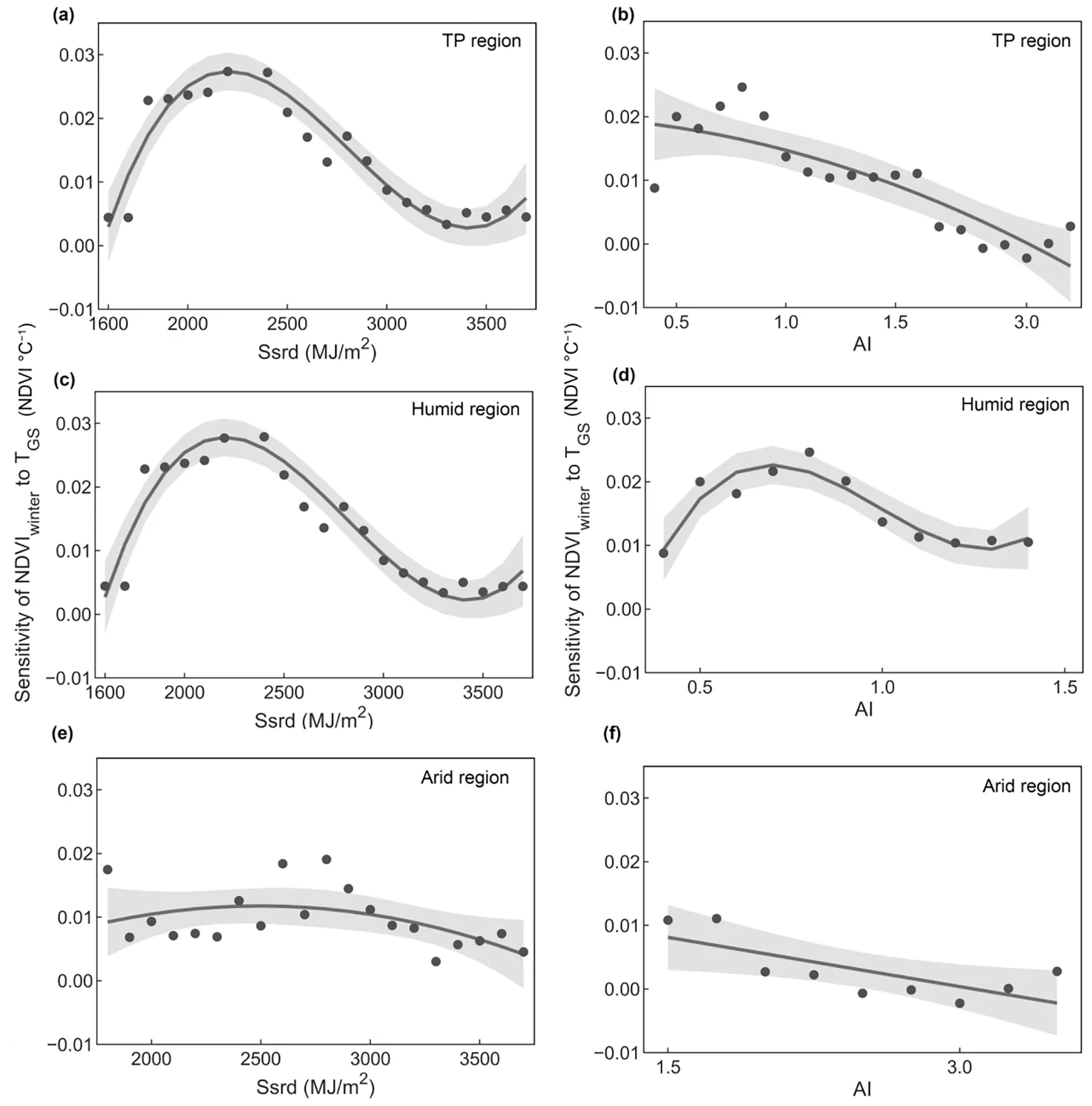
The variation of sensitivity of winter NDVI (NDVI_winter_) to growing season temperature (T_GS_) along key environmental gradients on the Tibetan Plateau. Panels show sensitivity of NDVI_winter_ to T_GS_ along downward shortwave radiation (Ssrd) and aridity index (AI) for **(a, b)** the entire Tibetan Plateau, **(c, d)** humid and semi-humid zones and **(e, f)** arid and semi-arid zones. Higher AI values indicate drier conditions.

We found that the factors affecting the sensitivity of vegetation to temperature exhibit subtle differences between arid and humid regions. Consequently, we conducted a more in-depth analysis of how the primary influencing factors, namely Ssrd and AI, impact spatial variation in vegetation sensitivity. On a regional scale, the sensitivity of NDVI_winter_ to T_GS_ initially increased, then decreased when Ssrd reached 2250 MJ/m^2^, before increasing again with further increases in Ssrd (**Figure 6a**). Additionally, the sensitivity of NDVI_winter_ to T_GS_ showed a linear relationship with AI, meaning that as AI increased, vegetation sensitivity decreased (**Figure 6b**). In humid zones, the sensitivity of NDVI_winter_ to T_GS_ in relation to Ssrd followed a similar trend to that observed at the regional level; however, its variation with AI deviated from the regional patterns (**Figures 6c, d**). Conversely, the sensitivity of NDVI_winter_ to T_GS_ in arid zones exhibited a similar relationship with AI as observed at the regional level and showed a parabolic relationship with Ssrd (**Figures 6e, f**).

## 4 Discussion

### 4.1 The underlying mechanisms of sensitivity of winter evergreen vegetation greenness to temperature

The sensitivity of vegetation to temperature demonstrates notable spatial variability, with a higher sensitivity in the southeast and lower sensitivity in the northwest (**Figure 3**). Notably, forests, especially mixed forests, displayed a heightened sensitivity in comparison to grasslands. We also observed the low regional average sensitivity in evergreen broadleaf forests. This may be attributed to the fact that annual precipitation in these regions often exceeds 4000mm, with local values surpassing 10,000mm, rendering vegetation growth fully water-saturated and temperature no longer a limiting factor. After accounting for static site heterogeneity by including pixelID as a random intercept in the LMM, the genuine temperature sensitivity becomes weak. Moreover, vegetation in humid regions also demonstrates a notably greater sensitivity and lower in arid zones. These findings consistently align with the results of previous research (Wang et al., 2024a; Wu et al., 2024).

The temperature sensitivity of evergreen vegetation on the Tibetan Plateau was primarily influenced by solar radiation and the aridity index (**Figure 4**), consistent with previous findings (Liu et al., 2025a; Wu et al., 2023). Although the random forest model revealed the dominant driving factors of vegetation temperature sensitivity, its overall explanatory power (R²) was relatively low, leaving nearly half of the variance unexplained. Such moderate performance is common in large-scale ecological modelling across alpine terrain (Wieder et al., 2017), as vegetation thermal responses are regulated by numerous unmeasured factors, including fine-scale soil and permafrost heterogeneity and multi-year environmental legacy effects (Buermann et al., 2018; Wieder et al., 2017). This unexplained variance stems from the intrinsic complexity of alpine ecosystems on the Tibetan Plateau rather than inherent defects in the random forest modelling framework.

The energy-related factors (e.g., growing-season solar radiation, temperature) determine vegetation physiological activity (Wang et al., 2024b). Sufficient heat generally improves vegetation metabolic efficiency and strengthens the response of winter greenness to temperature fluctuations, resulting in higher temperature sensitivity. However, in regions with excessive energy, extreme high temperatures may induce thermal limitation and reduce vegetation sensitivity to temperature (**Figures 5**, **6**). Water availability (e.g., aridity index, soil moisture) regulates temperature sensitivity by alleviating or exacerbating water stress. Further analysis showed that the dominant driving factors of temperature sensitivity differ between arid and humid regions. In humid areas, solar radiation acts as the key limiting factor, whereas the aridity index plays a decisive role in arid zones (**Figures 3**, **5**).

In humid regions with negligible water limitation, where forests are the dominant vegetation cover and the leaves are dense, increased solar radiation can raise temperatures and promote carbon assimilation and vegetation growth, as plants can efficiently utilize light under favorable moisture conditions (Fyllas et al., 2017; Liu et al., 2025a). Additionally, temperature sensitivity exhibited a nonlinear response to the aridity index in humid zones, with a turning point at an AI of 0.7–0.8, indicating a threshold response of evergreen vegetation to aridity under humid conditions and the vegetation does not simply linearly respond to changes in dryness, but rather plays a regulatory role. In contrast, aridity dominates the spatial variability of temperature sensitivity in arid zones. This is because water shortage imposes stronger physiological constraints on vegetation than light availability (Wang et al., 2021), and the effect of solar radiation is weakened due to plant physiological adaptations. Intense solar radiation may cause rapid light saturation by adjusting leaf nitrogen-phosphorus stoichiometry, often leading to photosynthetic inhibition (Fyllas et al., 2017; Wu et al., 2023). Furthermore, we observed a linear relationship between vegetation sensitivity and the aridity index in arid zones, which aligns with global patterns reported in previous research (Chen et al., 2024), indicating the significant role of aridity index on the vegetation in arid zones. This linear relationship highlights the critical role of aridity dynamics in regulating the responses of winter evergreen vegetation to climate change. In arid zones, grasslands are dominant land cover and narrow leaves reduce the leaf surface area exposed to the atmosphere, thereby minimizing transpiration water loss and improving water use efficiency. Previous studies have also verified the high sensitivity of vegetation to drought stress in arid regions (Tang et al., 2025). With global warming, arid regions are expected to experience intensified water stress, which may threaten the resilience and growth of evergreen vegetation. Meanwhile, climate warming may increase solar radiation and favor evergreen vegetation growth in humid zones. These results highlight the necessity of implementing region-specific ecological conservation strategies to address climate-driven ecosystem changes across different aridity zones on the Tibetan Plateau.

### 4.2 Uncertainties and implications

This study has certain limitations. Firstly, some uncertainty stems from inconsistencies in the spatial resolution of input datasets. Soil moisture was acquired at 1 km resolution, whereas climate variables including temperature and precipitation were sourced at 10 km. To enable spatial integration, all datasets were resampled to a common 500 m grid. While necessary for data harmonization, this resampling may attenuate fine-scale spatial heterogeneity, especially for precipitation, which varies sharply over the complex topography of the Tibetan Plateau, potentially introducing modest biases into the analysis. Despite these constraints, the adopted processing scheme represents a pragmatic compromise given currently available high-temporal-resolution climate products for the region. Furthermore, the strong spatial and temporal autocorrelation exists in the driving factors datasets. Although random forest model maintains stable predictive performance in the presence of strong multicollinearity among hydrothermal and topographic predictors (Breiman, 2001), this study only adopted static annual average environmental variables. Future research could integrate multi-source diversified data to capture fine-scale hydrothermal variations and further reduce modelling uncertainty.

Additionally, the temperature sensitivity analysis utilizes a linear regression framework, which is widely adopted in vegetation-climate change research due to its interpretability and direct quantification of sensitivity parameters. However, considering that vegetation-climate interactions often exhibit nonlinear dynamics, future investigations will add more driving factors and explore advanced modeling approaches. Although random forest model effectively captures complex nonlinear statistical associations between environmental factors and vegetation temperature sensitivity, the model cannot identify causal mechanisms. All factor rankings and marginal response curves merely reflect correlation patterns rather than direct causal effects. In the subsequent stage, the interaction effects and mechanisms of the driving factors will be enhanced for exploration.

## 5 Conclusions

This study reveals significant spatial heterogeneity in the sensitivity of winter NDVI to growing season temperature across the Tibetan Plateau, with mixed forests and humid regions exhibiting the highest sensitivity. The most important predictors in the random forest model of this spatial variability are downward shortwave radiation and the aridity index, although topographic factors play a more prominent role in arid regions. Nonlinear relationships between key driving factors and NDVI_winter_ sensitivity highlight the complexity of vegetation response to temperature, with threshold-like responses observed for DEM and Ssrd. The sensitivity of NDVI_winter_ to T_GS_ initially increases and then decreases, before increasing again, while it declines with increasing AI. These findings underscore the importance of considering regional-specific environmental controls when assessing vegetation sensitivity to temperature, providing valuable insights for ecological management and climate change adaptation strategies in the Tibetan Plateau and similar regions.

## Data Availability

The original contributions presented in the study are included in the article/**Supplementary Material**. Further inquiries can be directed to the corresponding author.
