# Supplementary material for: Spatial patterns and drivers of temperature sensitivity of winter evergreen vegetation on the Tibetan Plateau
Source: Front Plant Sci. 2026 Aug 20;17:1891108. doi: 10.3389/fpls.2026.1891108 (PMC13538431; doi:10.3389/fpls.2026.1891108)
Supplement: Supplementary file 1 [file SupplementaryFile1.docx]

Supplementary Figures for

Spatial Patterns and Drivers of Temperature Sensitivity of Winter Evergreen Vegetation on the Tibetan Plateau

Jinxia Lv ^1^,Chun Dong ^1^*, Guangyong Li ^2^, Xiaohuang Liu ^3^, Rui Zhang ^4^ and Rong Zhao ^1^

^1^ Chinese Academy of Surveying and Mapping, Beijing 100036, China

^2^ Beijing Forestry University, Beijing, 100083, China

^3^ Laboratory of Coupling Process and Effect of Natural Resources Elements, Ministry of Natural Resources, Beijing 100055, China

^4^ State Key Laboratory of Cryospheric Science and Frozen Soil Engineering, Key Laboratory of Remote Sensing of Gansu Province, Heihe Remote Sensing Experimental Research Station, Northwest Institute of Eco-Environment and Resources, Chinese Academy of Sciences, Lanzhou 730000, China

*****	Correspondence: [dongchun@casm.ac.cn](mailto:dongchun@casm.ac.cn)

This file includes 6 figures and 1 table.

Figure S1 Spatial distribution of partial least squares regression coefficients between monthly mean temperature and winter NDVI from 2000 to 2021.

Figure S2 Spatial distribution of variable importance for partial least squares regression coefficients between monthly mean temperature and winter NDVI from 2000 to 2021.

Figure S3 Spatial distribution of partial least squares regression coefficients between monthly mean precipitation and winter NDVI from 2000 to 2021.

Figure S4 Spatial distribution of variable importance for partial least squares regression coefficients between monthly mean precipitation and winter NDVI from 2000 to 2021.

Figure S5 Distribution of the sensitivity of winter NDVI (NDVI_winter_) to growing season temperature (T_GS_) at 0.05 significance level.

Figure S6 The distribution of root mean squared error (RMSE) and coefficient of determination (R^2^) of random forest model across 100 independent repetitions for (a) Tibetan plateau (TP) region, (b) humid region and (c) arid region.

Table S1 The Mean and standard deviation of variable importance score (VIP) for driving factors across 100 replicate experiments of random forest model.

| Variables | TP region | | | Humid region | | | Arid region | | |
| --- | --- | --- | --- | --- | --- | --- | --- | --- | --- |
|  | Mean VIP | Standard deviation | Coefficient of variation (CV) | Mean VIP | Standard deviation | Coefficient of variation (CV) | Mean VIP | Standard deviation | Coefficient of variation (CV) |
| Ssrd | 0.1616 | 0.0006 | 0.0039 | 0.1686 | 0.0007 | 0.0041 | 0.1025 | 0.0012 | 0.0113 |
| AI | 0.1491 | 0.0015 | 0.0102 | 0.1526 | 0.0026 | 0.0171 | 0.1322 | 0.0008 | 0.0057 |
| DEM | 0.1398 | 0.0004 | 0.0032 | 0.1367 | 0.0007 | 0.0051 | 0.1795 | 0.001 | 0.0055 |
| SM | 0.1262 | 0.0004 | 0.003 | 0.125 | 0.0005 | 0.0039 | 0.1338 | 0.0009 | 0.0067 |
| Aspect | 0.1213 | 0.0003 | 0.0024 | 0.1179 | 0.0003 | 0.0027 | 0.1503 | 0.0008 | 0.0055 |
| Pre | 0.1042 | 0.0008 | 0.0073 | 0.1109 | 0.001 | 0.0094 | 0.1029 | 0.0011 | 0.0111 |
| Tmean | 0.1012 | 0.0017 | 0.0169 | 0.0934 | 0.0027 | 0.0286 | 0.0869 | 0.0014 | 0.0161 |
| Slope | 0.0968 | 0.0002 | 0.0023 | 0.0949 | 0.0003 | 0.0029 | 0.1119 | 0.0007 | 0.0060 |

Abbreviations: Ssrd: multiyear mean of cumulative downward shortwave radiation in growing season during 2000-2021; Pre: multiyear mean of cumulative precipitation in growing season during 2000-2021; Tmean: multiyear mean of average temperature in growing season during 2000-2021; AI: multiyear mean of annual aridity index during 2000-2021; SM: multiyear mean of soil moisture of 0-100 cm during 2000-2021.
